# Migration motives and integration of international human resources of health in the United Kingdom: systematic review and meta-synthesis of qualitative studies using framework analysis

**DOI:** 10.1186/s12960-018-0293-9

**Published:** 2018-06-27

**Authors:** Latha S. Davda, Jennifer E. Gallagher, David R. Radford

**Affiliations:** 1 King’s College London Dental Institute, University of Portsmouth Dental Academy, The William Beatty Building, 1 Hampshire Terrace, Portsmouth, PO1 2QG UK; 2 Dean for International Affairs, Newland Pedley Professor of Oral Health Strategy, King’s College London Dental Institute, Denmark Hill Campus, Bessemer Road, London, SE5 9RS UK

**Keywords:** Migration motives, Integration barriers and facilitators, Internationally trained nurses, International medical graduates, International dental graduates

## Abstract

**Objective:**

The aim of this review was to examine the migration motives, the barriers to and facilitators of integration of international dental graduates, compared with nurses and doctors in the United Kingdom.

**Methods:**

Electronic databases Ovid MEDLINE, EMBASE, PubMed, Web of Knowledge and OECD publications were systematically searched for English language publications from January 2000 to January 2017. A total of 31 qualitative studies were selected and quality appraised and meta-synthesis of the qualitative data was carried out using framework synthesis. The Preferred Reporting Items for Systematic Reviews and Meta-Analyses (PRISMA) guidelines were applied to present the findings.

**Results:**

There were no studies on migration motives and one study on integration experiences of international dentists in the UK. The nursing literature had the highest volume and quality of evidence on nursing workforce, whilst there was limited literature on international doctors in the UK.

Migration of health professionals to the UK is determined by personal and professional factors, together with source country-specific and UK drivers. Active recruitment, post graduate training and financial gain act as strong common macro, meso and micro drivers that perpetuate migration into the UK, but the extent to which each of these drivers influence nurses’ and doctors’ migration is different.

Integration experiences for international nurses and doctors differed based on their source country experiences and the work environment they entered. Nurses reported a wider knowledge and skills gap, more multi-level discrimination and less career progression compared to the doctors. The migrants’ integration experiences depend on their cultural awareness, discrimination exposure, English language and communication skills, social and professional support networks, social integration and personal attributes.

**Conclusion:**

Migration of international health professionals is motivated by macro, meso and micro drivers at the international, national, professional and personal levels. The UK has strong common macro pull factors which attract nurses, doctors and dentists and may impact on the effectiveness of policies to restrict their migration. The integration experiences of nurses and doctors differ and further research is required to understand the integration experiences of dentists, in order to retain these professionals by tailoring policies to each of these professions.

**Electronic supplementary material:**

The online version of this article (10.1186/s12960-018-0293-9) contains supplementary material, which is available to authorized users.

## Background

International migration of human resources for health (HRH) affects individuals themselves and has an impact on both the source and destination countries’ health systems by affecting service provision, health policies [1, 2], workforce planning [3], training and education [4], and the social and economic development of these countries. It is one of the underlying reasons behind the global HRH crisis resulting in global health inequalities [1]. The World Health Organization (WHO), the Health Worker Migration Policy Initiative (HWMPI) and the Global Health Workforce Alliance (GHWA) developed a Global Code of Practice on recruitment of international health personnel, which was adopted by 193 member states in May 2010 [2]. The implementation of the code has remained partially successful both globally and in the United Kingdom [3]. The UK’s historic reliance on international HRH has created established organisational, cultural, professional and social networks that facilitate migration, thereby decreasing the UK’s ability to act on the WHO’s Global strategy on HRH to half the reliance on foreign workforce by 2030. This is despite policies for increasing domestic workforce and better use of skill mix [4]. Therefore, HRH mobility is of high relevance to the UK, whilst it is actively developing policy and regulatory interventions to reduce its reliance on an external workforce [5].

In sharp contrast, NHS England’s (2016) workforce planning, along with other measures, aims to recruit 500 international doctors through an international recruitment campaign to meet the targets of extra 5 000 doctors by 2020 [6]. The National Health Service (NHS) employs more than 1.7 million people across England, Scotland, Wales and Northern Ireland [7]. Data for new registrants to the UK showed that the percentage of internationally trained nurses increased from 11% (*n* = 2, 121) in 1993 to 53% (*n* = 16, 155) in 2001/2002 and decreased to 30% (*n* = 8, 785) in 2016 [8, 9]. International doctors increased from 37% in 1990 to 71% (*n* = 11, 106) of new registrants in early 2003 and subsequently decreased to 41% (*n* = 5, 263) in 2016 [7, 10]. Similarly, the proportion of international dentists has increased from 38% (*n* = 524) in 2001 to 66% (*n =* 1, 481) in 2005 and then decreased to 35% (*n* = 795) in 2016 [61]. Retention of these health professionals is important; however, since the UK’s vote on the EU referendum, trends suggest more EU nationals are leaving the NHS [9]. This is compounded by the increase in the ageing domestic workforce leaving the profession, decreased job satisfaction due to decreased staffing, pay freeze, emigration and migration of the workforce into the private sector [11]. The UK’s exit strategy from the European Union ‘Brexit’ may impact further on the retention of European HRH.

The aim of this systematic review was to examine the migration motives, barriers to and facilitators of integration of international nurses, doctors and dentists to the UK, to inform policies on international recruitment, retention of workforce and identify future workforce planning research gaps. Along with retention of this international workforce in the NHS, their integration, training and support are required to maintain patient safety. Understanding barriers to and facilitators of integration for this mobile workforce will help to inform future research into suitable adaption processes.

## Methods

### Search strategy

A review protocol was designed (Table 1) following the Preferred Reporting Items for Systematic Reviews and Meta-Analyses (PRISMA) guidelines [12]. Electronic databases Ovid MEDLINE, EMBASE, PubMed, Web of Knowledge and OECD publications were searched for English language publications from 1 January 2000 to 31 January 2017. The search words were used by combining words using Boolean operators (Table 2). Original research, case studies and reports with qualitative data reporting on migration motives and/or integration of health care professionals were included. A sample of search conducted is available (see Additional file 1).

**Table 1:** The Population, Issues, Context, Outcomes, Study design of the systematic review

Population	Internationally trained nurses (nurses), International medical graduates (doctors), International dental graduates (dentists) working in the UK
Issues	Migration and integration of the above populations in the UK
Context	Working in the health care sector
Outcomes	1. Migration motives of the nurses, doctors and dentists 2. Barrier and facilitators of Integration of the above populations
Study design	Eligibility criteria was set for selection of qualitative and mixed method papers Information sources selected Search strategy designed Data collected Quality of papers appraised with CCAT Framework analysis used for meta-synthesis of data

**Table 2:** Search terms utilised in the review

Column A	Column B
Internationally trained nurses, overseas trained nurses, foreign nurses International medical graduates, internationally trained doctors, foreign doctors, overseas doctors International dental graduates, overseas dental graduates, foreign dentists, overseas dentists International healthcare workers, International health care professionals, foreign healthcare workers, overseas trained health care professionals	Migration, migration motives Integration Adaptation Support Career aspiration Job satisfaction Performance Discrimination

Terms and their abbreviations from column A were combined with those in column B

The lack of universally accepted definitions to the search terms was recognised and the variation in their use in the titles resulted in non-identification of relevant papers which was overcome by hand searching the references in key papers. The papers were initially selected by means of their titles. Abstracts of all the selected papers were read by two authors (LSD, DRR) and 137 papers were shortlisted. An eligibility checklist (Additional file 2) was used to finalise the list. The papers reporting primary qualitative data on migration motives, barrier to and facilitators of integration of international nurses, doctors and dentists, were included. This review was undertaken as part of a study on international dentists in the UK, examining the limited dental literature and drawing on the more extensive health care literature for nursing and medicine. Whilst doctors and dentists have some similarities in relation to their status, all the three groups have similarities in their educational components, registration pathways and employment in the NHS, when migrating to the UK.

### Quality assessment, data extraction and analysis

All papers were quality assessed using Crowe’s critical appraisal tool (CCAT) [13]. A sample (8/31; 25%) of the papers was assessed independently by a second researcher (DRR) for calibration. Data extraction involved the primary researcher (LSD) reading the selected papers several times and creating annotated summaries of sample characteristics, research methodology, data collection instruments, data analysis, outcomes reported and emerging themes. Qualitative data for migration motives and integration were selected from the results and discussion sections of papers to create annotated summary sheets (see Additional file 3). The risk of bias in each study was assessed in line with the CCAT tool, including consideration for potential bias in sampling, declaration of the relationship between the researcher and participants and interpretation of results.

Meta-synthesis of the qualitative data was carried out using framework synthesis which has successfully been used to inform policy and practice in health sciences where a conceptual framework is used to map and analyse the data [14]. Framework synthesis is appropriate for heterogeneous data and is based on an epistemological standpoint of critical realism that the knowledge of reality is mediated through our perceptions and beliefs [15], whereby a priori coding is derived from literature and new codes are added as themes emerge.

The framework of Young’s model of macro- (global and national factors), meso- (profession led factors) and micro-level (personal factors) drivers of migration [16] was used to analyse the migration motives. Barriers to and facilitators of integration were identified in each study along with the phase at which they were operating. Each barrier and facilitator was then examined to explore the differences between groups, and overarching themes were derived across all groups. All three authors (LSD, JEG and DRR) were involved in agreeing the framework for the analysis and reaching consensus on the overarching themes emerging from the data.

## Results

**Fig. 1:**
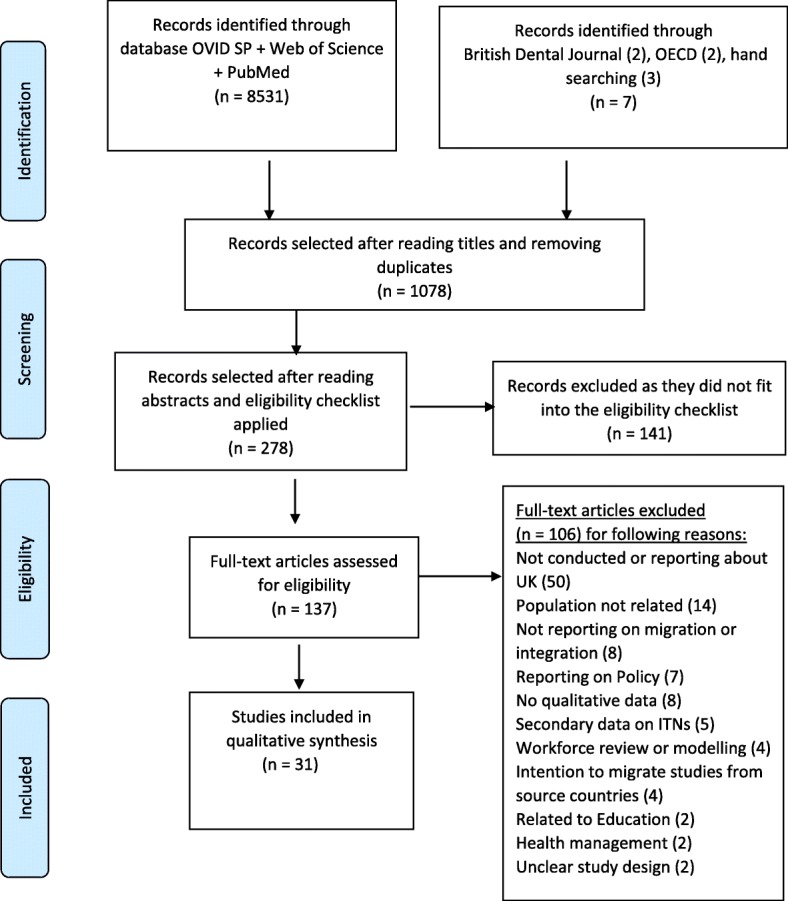
Process of selection of papers for systematic review (PRISMA 2009 flow diagram)

The number of papers screened, assessed for eligibility and included in the review, together with details of exclusions, is presented in the PRISMA flow diagram (Fig. 1). There were no studies published on the migration motives of dentists to the UK and just one study on a pre-registration training programme relevant to the integration themes [17].

A summary of the study methods, type of participants, methodological approach, sample sizes, sampling process, study settings and the country of primary qualification in the 31 papers selected for this review is presented in Table 3. Reporting qualitative systematic review using PRISMA guidelines showed that some items on the reporting checklist were applicable to quantitative studies and using qualitative reporting tools [18, 19] would have been an alternative approach.

**Table 3:** Overview of the 31 papers selected for systematic review

Study information	Study Methods	Type of participants
Year of publications	Data collection methods *n* = studies	Professional groups *n* = studies
1 January 2000 to 31 January 2017	Interviews *n* = 24 Questionnaire surveys *n* = 8 Focus groups *n* = 6 Field Observations *n* = 3	Internationally trained nurses (Nurses) *n* = 19 International medical graduates (Doctors) *n* = 8 International dental graduates (Dentists) *n* = 1 International nurses and doctors *n* = 3
Country	Methodological approach	Total sample size *n* = participants
UK *n* = 27 UK + EU countries *n* = 1 UK + EU + International *n* = 2 UK + International (non EU) *n* = 1	Explorative qualitative *n* = 7 Phenomenology *n* = 6 Mixed method *n* = 5 Interpretive phenomenology approach *n* = 4 Case study *n* = 3 Grounded theory *n* = 2 Ethnography *n* = 1 Not clear *n* = 3	Nurses *n* = 2 202 Doctors *n* = 517 Dentists *n* = 5
Settings	Sampling	Country of primary qualification represented
NHS hospitals *n* = 17 Nursing/care homes *n =* 4 Training programmes *n =* 3 Mixed setting *n =* 3 Primary care *n =* 2 Adaptation/induction programmes *n =* 2	Purposive/theoretical *n =* 16 Random *n =* 8 Snowballing *n =* 4 Convenience *n =* 2 Not clear *n =* 1	Outside EU: Afghanistan, Bangladesh, Caribbean islands, China, Cuba, Egypt, Germany, Ghana, India, Iran, Iraq, Jordan, Mauritius, Nepal, Nigeria, Pakistan, Philippines, Russia, Sierra Leone, South Africa, Sudan, Syria, United Arab Emirates, USA, Zimbabwe EU: Austria, Estonia, France, Germany, Greece, Hungary, Ireland, Italy, Lithuania, Malta, Poland, Portugal, Romania, Slovenia, Spain, Netherlands

### Quality rating and risk of bias

The quality of the studies was assessed in line with CCAT score resulting in high (≥ 31–40), moderate (21–30) and low (≤ 20) scores. Almost half (*n* = 15) out of the 31 studies were scored high and a similar number (*n* = 14) as moderate, with only two papers scored as low. The second reviewer (DRR) scored 8 out of the 31 papers (25%). Percent agreement between the reviewers was 0.78, with 100% inter-rater agreement with kappa score of 1 for the overall score for each paper. The agreement was high for study design, sampling, data collection, ethical matters and results and the differences arose in evaluating the discussion section which may be due to second rater having more experience in evaluating qualitative papers. Papers were scored lower when the abstracts did not reflect the outcomes and when there was no clarity on the rationale of the chosen research design or methodology or researchers’ philosophical approaches to qualitative research.

The risks involved in qualitative studies are, of selection bias in sampling and recruitment, interviewer bias based on their training and background and recall bias if a phenomenon is studied. The papers selected in this review were scored high for the design if bias had been acknowledged and results interpreted accordingly.

### Migration motives of nurses and doctors to the UK

The decision to migrate to the UK is made by an individual on a personal level; however, the how, why, where and when to migrate appears to be determined by a complex interplay of professional, local, national and international drivers (Table 4) [16, 20–22]. The interplay of factors is dependent on the type of profession, source country training and working environment, individual’s career aspiration and destination country’s pull factors [20].

**Table 4:** Migration motives of international doctors and nurses to the UK

Macro-level driver	International and national factors that exert influence across all international labour markets and also affect the health system dynamics [16, 20]		
	Themes and descriptions	Codes	References to clarify the source of the codes
UK based	Health system factors (these are factors related to the UK health systems including NHS)	Active recruitment Employment opportunities in the NHS Safety and security of NHS Established networks Support offered for relocation/induction Permit free training	Active recruitment [21, 25–31] Employment opportunities in the NHS [26, 27, 32–34] Safety and security of NHS [25, 26, 35, 37–39] Established networks [29, 31]
	Economic factors	Ability to remit money Strength of pound in global economy	[31, 33, 35, 39]
	Political factors	Bilateral agreement UK referendum vote to leave EU Safety for family/self, fleeing violence Ease of obtaining right to remain Ease of British Citizenship Ease of movement to the UK from EU	Bilateral agreement [25, 29, 40]
Source country based	Health system	Unemployment Underemployment Poor salaries Poor working conditions Overproduction of nurses and doctors	Un/underemployment Poor salaries [41] Working conditions [27, 33]
	Economic factors	Global recession Devaluation of money Changes to remuneration Remittance to home country	Global recession [30] Devaluation of money [25] Changes to remuneration [41] Remittance to home country [38, 39, 42]
	Social factors	Corruption in everyday life	Corruption in everyday life [26, 29, 33]
	Political factors	Immigration policies Bilateral agreements Colonial connections	Immigration policies Bilateral agreements [8, 34]
Meso-level	Professional-specific factors (e.g. education/training, job conditions) that frame perceived opportunities in a given occupational sector		
UK	Training opportunities	Desire to gain postgraduate training Desire to gain postgraduate qualification Desire to learn the state of the art in the profession Status of gaining UK qualifications and training	Training opportunities [26, 27, 32–34]
	Employment opportunities	Desire to experience working in a different environment	Experience a different work environment [35, 50, 51]
	Career progression opportunities	Opportunities to gain clinical experience through short-term employment Opportunity for research Opportunity for networking	Opportunities to gain clinical experience through short term employment [43, 48]
Source country	Training and employment opportunities	Shortage of postgraduate training opportunities Shortage of posts in a particular speciality/profession Natural progression of training	[26, 49, 53]
	Career progression opportunities	Lack of promotion	[30, 41, 49, 50, 53]
Micro-level	Individual circumstances and attitudes through which macro- and meso-level drivers are viewed by individuals therefore influencing individuals’ migration decision		
	Personal fulfilment	Desire for life change Adventure Better quality of life	Desire for life change Adventure [52] Better quality of life [25, 29, 37, 40, 46, 48]
	Financial gain	Financial gain for self Financial gain for family Financial gain for extended family Desire to increase comparative income	Financial gain for self and family [26, 27, 29, 33, 35, 38, 42, 48, 55]
	Family factors	Better quality of life for family Better education for children Desire to give children the cultural experience Partners decision to work in the UK Travel to the UK to marry Travel to the UK to escape marriage	Better quality of life for family Better education for children Desire to give children the cultural experience [25] Partners decision [35, 50, 51]
	Networks	Access to social networks in the UK Access to professional networks in the UK Influenced by family mentor Influenced by professional mentor	Access to networks and mentors [37, 38, 42, 51] Role of mentor or mentoring [28]
	Language	Knowledge of English language Desire to improve English language	[25, 29, 39, 47, 48]
	Window of opportunity	One off opportunity	[25]
	Migrating stepping stone	Working in the UK is seen as a potential stage in onward migration, primarily to the United States, Gulf countries and Australia	[27]

#### Macro drivers

The UK health system had major pull factors for both nurses and doctors for career advancement [22–29], training [22, 23, 26, 27, 30, 31], safety and security of the NHS [22–24, 26, 27, 30, 31] and economic benefits [23–26, 29–35]. This was promoted by active recruitment, relocation packages for nurses and doctors from the EU [22, 30, 32]. Established migration networks [22, 34], social and political factors of UK’s tolerant society and commonwealth connections [25, 34] were other macro drivers. The push factors of source country were corruption [24, 25, 34], poor health infrastructure [25, 30] and lack of career advancement and training [27, 30, 35]. Access to the internet in the source countries facilitated nurses’ migration, by providing insight into wider nursing practices and online application to registration and employment in the UK [23, 27].

#### Meso drivers

Meso drivers were professional factors of perceived opportunities for career advancement [23–29] and training [23, 26, 27, 30, 31], which were important for nurses and doctors. The nurses felt valued as team members in the UK, compared to their source countries, and doctors felt more secure working in larger teams.

#### Micro drivers

The key micro drivers that attracted HRH to the UK were as follows: first, economic factors of financial gain for self and family [23–26, 29–32, 34, 35], and, second, personal factors including personal fulfilment, desire for life change, better quality of life, better education for children and the ability to speak the English language [22, 24, 27, 34, 35]. Amongst nurses, economic factors were particularly important [24, 30, 32], whilst for doctors, personal factors including knowledge of existing networks, personal fulfilment, desire for life change and a better life for their family were important [22, 33–36].

**Fig. 2:**
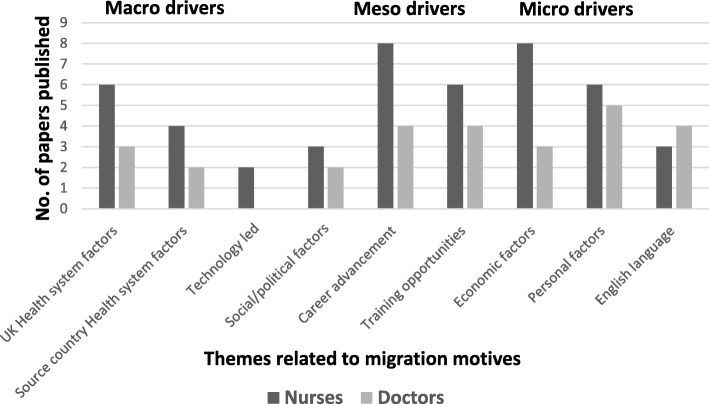
Themes related to migration motives of international nurses and doctors

Comparison of the themes on migration motives to the UK amongst nurses and doctors showed that there were many similarities and some differences in the migration drivers for each group (Fig. 2). Active recruitment was common for both groups, but bilateral agreements played a major role in ‘batch recruitment’ of nurses compared with doctors. The key differences in the meso drivers reported by nurses were poor salaries and shortage of postgraduate training or progression in their country  of origin, whilst doctors reported working in a different environment and gaining a post graduate qualification as important. Whilst there were not many differences in the range of personal drivers affecting both nurses and doctors, nurses were driven more by financial gain and gave less importance to English language skills. In marked contrast, as there were no studies published on the migration motives of dentists to the UK, their motives remain unknown.

### Barriers to and facilitators of integration

**Fig. 3:**
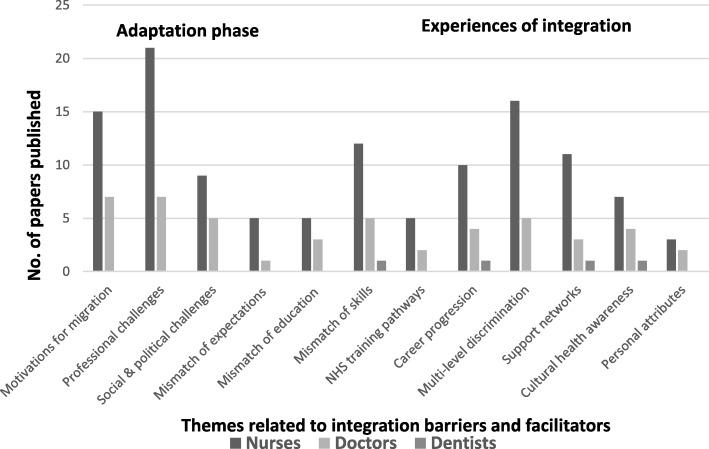
Themes for barriers to and facilitators of integration for international nurses, doctors and dentists

Research on integration of international HRH has focused on the migrants’ life journeys before and after the entry to the UK [23, 24, 27, 29, 37], their transition to work [33], their induction/adaptation/support training [17, 22, 33, 38–40], career aspirations, progression and job satisfaction [27, 28, 37, 41]. The main themes identified during adaptation and integration for the three groups of migrants are presented in Fig. 3.

The barriers to and facilitators of integration were influenced by where the participant was in their journey, the type of profession, and individuals’ experiences of integration as summarised in Table 5, which lists the themes and their references.

**Table 5:** Migrants’ journey and the barriers to and facilitators of integration of international nurses, doctors and dentists

Main theme based on migrants’ journey	Sub-themes [References]	Barriers to integration	Facilitators of integration
Adaptation	Motivation for migration [16, 20, 26, 28, 30–32, 34, 35, 37, 39, 40, 43, 44, 48, 49, 53]	Motivations for migration not met	Motivations for migration met
	Professional challenges [17, 20, 26, 29, 32, 33, 35, 39–41, 44, 45, 49–53]	Registration barriers Employment barriers Lack of professional mentors or networks	Recognition and transferability of qualifications and training Less duration of time for registration and employment Support from professional mentor or network
	Social and political challenges [20, 26, 35, 37, 40, 41, 43, 48–50, 52]	Lack of social and family network Immigration barriers	Pre-existing social and family networks Right to remain or British citizenship
	Mismatched expectations [30, 32, 35, 39, 40, 49–51, 53]	Mismatched expectations between migrants, managers and organisations	Prior knowledge of destination country and systems Ability to communicate and accept diverse views balance of autonomy and scrutiny
Career progression	Mismatch of education [26, 32, 33, 37, 41, 42, 49]	Non-recognition of training received in source country Gaps in education caused by migration Variation in education between countries	Adaptation or transitional courses improved education Translation of source country training into UK workplace and its acceptance by employers
	Mismatch of skills [17, 24, 26, 32, 33, 35, 37, 40–42, 45, 46, 49–52]	Good verbal and non-verbal communication skills Good English language skills Good technology-related skills Good interpersonal skills	Self-awareness Opportunities for training in listed skill
	NHS training pathways [26, 28, 34, 39, 40, 47, 48, 55]	Lack of access to NHS training and NHS jobs National and regional variation reducing career choice	Equal opportunities based on merit
Experiences of integration	Multi-level discrimination [20, 28–32, 34, 35, 39, 42–45, 47, 48, 50, 55]	Discrimination from patients, colleagues, managers and organisations Exploitation in care homes across EU Discrimination in pay Discrimination in progression	Equality and diversity training for all Organisational policy and frameworks to promote equal opportunities Self- actualisation, resilience, focus on long-term goal Moving away from ethnocentric views
	Support networks [24, 30, 32, 34, 37, 41, 42, 45, 47–52]	Lack of personal, professional, social and organisational support networks Lack of mentors and ability to mentor Insensitivity to interpersonal dynamics	Access to formal or informal, professional or social, individual or organisational networks for support Ability to mentor and be mentored Positive feedback loops from patients, colleagues, managers Sensitivity to interpersonal dynamics
	Cultural health awareness [24, 32, 34, 35, 37, 41, 43, 45, 48, 49]	Social isolation and lack of international exposure Unwillingness to exchange of knowledge and skills Struggle between self, social and professional identity	Knowledge of behaviours and ability to adapt Interactional styles Health literacy related to the country, population and beliefs
	Personal attributes [17, 30, 32, 47, 48, 50]	Lack of flexibility Low self-esteem Lack of self-awareness Lack of emotional maturity Insensitivity to interpersonal dynamics Lack of insight and reflection Self-blame and negative psychosocial wellbeing	Personal traits of adaptability, resilience, lateral thinking Sensitivity to interpersonal dynamics Entrepreneurship Focus on long-term goals Positive psychosocial well being

#### Initial adaptation

The adaptation phase was the most difficult phase for migrants as they faced the challenges of registration examinations, recognition of previous education and skills and securing employment in the NHS. This was compounded with financial worries, immigration difficulties and social isolation. The delays inherent in the registration and employment process led to deskilling, devaluation and demotivation. This was particularly reported by nurses who worked in the care home sector during the transitional period. The converse, i.e. less time between entry to the country and employment, recognition of source country education and skills, support from social and professional networks along with presence of family, facilitated integration. This was more likely the case for migrant professionals from EU to the UK. Migrant nurses were mostly female, reflecting the nursing workforce globally and gender can be a confounding factor in analysing the interpretations on how the participants perceive their journeys and work environment [31, 37]. Female doctors found it difficult to find jobs and were more likely to be ‘re-domesticated’ with longer gaps in employment [37], and less satisfied with their career progression compared to nurses [38].

#### Career progression

Nurses and doctors reported that once in employment, the mismatch of education, skills and expectations between migrants, managers and employers resulted in reported dissatisfaction, devaluation and deskilling leading to difficulty in integration. Lack of verbal and non-verbal communication skills, interpersonal skills, English language skills and technology-related skills influenced their career progression. Nurses specifically reported cultural displacement, variation in communication and technical skills, fear of speaking out and thus becoming invisible in the system.

Amongst doctors, non-recognition of qualifications and skills, career stagnation in non-training posts and language barriers decreased job satisfaction whilst better work-life balance, security of a salaried post, working in large teams increased job satisfaction [22]. During post-graduate foundation training, UK graduates were reported as being better at history taking and some communication tasks whilst migrant doctors performed better in clinical skills [33]. Educational supervisors noted that international doctors were unfamiliar with the use of portfolios and reflective practice, clear differences in communication, whereby they were more directive with patients and more subservient to senior doctors, which could be misinterpreted as lack of confidence [33]. These differences may be a reflection of the training and professional cultures of the source countries.

#### Experiences of integration

Integration of the migrant workforce was influenced by the exposure to discrimination at different levels [28–32, 34, 35, 39, 42–47], the awareness of cultural and health diversity [22, 24, 34, 35, 37, 41, 43, 44, 48, 49], their formal and informal support networks [24, 30, 32, 34, 37, 41, 42, 45–52] and their personal attributes [27, 30, 32, 34, 47, 48] Multi-level discrimination by patients, colleagues and managers featured strongly in the nursing literature. There was perceived discrimination in pay, employment, training and progression towards nurses from Asia and Africa compared to those from New Zealand, Australia and America [26, 42, 50]. Taylor in 2005 [41] reported that UK colleagues saw ‘non-white’ or those whose first language was not English as ‘overseas’ and not others, suggesting an unconscious bias based on ethnicity and spoken English. Victims of discrimination reacted either by ignoring it and focusing on their career development or they accepted it and stopped aspiring [27, 51]. Doctors from the EU reported discrimination in training, job opportunities and some discrimination from colleagues but none from patients [35, 38], whilst ethnic minority doctors from Europe found the UK more welcoming [34].

Lengthy registration processes were the main barrier to integration for all HRH migrating to the UK. Nurses reportedly found it easier to find a job in the care home sector whilst waiting to be registered, but the doctors struggled to get employment. Once they obtained jobs, the nurses reported difficulty in adaptation due to gaps or mismatch in knowledge and skills, whilst migrant doctors reported being knowledgeable, but had gaps in communication skills similar to dentists [17, 33]. Amongst nurses, factors such as longer duration of stay, easier route to registration, early employment, professional support and mentoring, understanding and valuing diversity, enhanced their integration [23, 25, 27, 31, 51]. Equality and diversity training highlighting cultural variations, improved integration [27]. There is very little knowledge on integration experiences of international dentists working in the UK.

## Discussion

This systematic review highlights the complexity and the differences in health professionals’ motivation to migrate to the UK and their integration [7, 11, 16, 20, 43, 48]. Whilst migrants describe their motivations as mainly driven by micro and meso factors, there are strong established macro factors including active recruitment and bilateral agreements between countries, which facilitates migration.

A key meso driver for HRH migrating to the UK was post graduate training opportunities. For doctors, gaining post graduate qualification was more important than nurses, as this would improve their career progression both in the UK and in the source country should they return. Doctors reportedly earned well in comparison to the rest of source country population and therefore financial gain, although important, was not the main micro driver for migration as compared to the nurses.

Integration studies using cultural frameworks of Pilette’s theory of adjustment (1989) [29], and socio-cultural theories describing phases of acquaintances, indignation, conflict resolution, and acculturation [53], are useful to understand how individuals react when they enter new cultures. Integration experiences of individuals are dependent on the process of learning, developing social and professional identity, understanding the local work place cultures, cross-cultural awareness [38] and improved interpersonal communications. Illing [33] and Hofstede’s model (2001), explains the different distances individuals have to move to make the transition from their training culture to the new work place culture and hence the variations in the adaptation experiences of the doctors and nurses are based on their source country training and personal attributes. Similar to a review conducted in Australia, most international professionals seemed to struggle during the transition from training to work and found it difficult to integrate when they did not have access to adaptation programmes, thus, stressing the importance of investment in such programmes to improve retention and patient safety [54].

The nurses in the UK reported multi-level discrimination, whilst doctors reported less individual and more institutional discrimination resulting in career stagnation. This may be as a result of nurses being in direct contact with patients for longer periods of time and the struggle to control the work environment in hospitals with established hierarchy based on race and gender, whilst doctors had more control on their work environment [27]. Migrant nurses were more likely to set up informal professional and social networks during the transition period, to deal positively with discrimination [46, 52] and get social support, as many lived in the UK without their families, due to immigration restrictions. During integration, whilst there were obstacles in terms of verbal and non-verbal skills, international doctors and dentists felt no knowledge gaps compared to the nurses [17, 30, 52]. That may be explained by the fact that all non-EU doctors and dentists have to take the registration examinations set by the respective registering bodies, which tests their knowledge rigorously. A similar competency test was introduced for nurses in 2014, which may have improved the knowledge gap. Further research is needed into the role of global interconnectedness, social media and virtual networks in the context of HRH migration and integration. More research needs to be done on the effect of migration on migrant nurses and dentists performance, as doctors’ performance was linked to their move to the UK and their experiences of social and cultural isolation, disorientation, financial hardship, language difficulties and their ability to understand multi-disciplinary team working [33], illustrating the need for comparative studies.

It is important that organisations involved in the registration and employment of international HRH, have fair systems in place to recognise HRH international education and training to improve their integration [33, 55]. Retention can be improved through enhancing integration by providing training in equality and diversity [47]. If bespoke support programmes are not put in place to improve integration, dissatisfaction with the system, deskilling, economic and political uncertainties (Brexit) could lead to onward migration of HRH to other English speaking destination countries or repatriation. Emigration of UK-trained doctors and nurses and ageing domestic workforce could further reduce the overall health care workforce in the UK, making international recruitment unsustainable, similar to the situation in South Africa and Republic of Ireland [56].

There have been systematic reviews of international HRH in OECD countries, Australia, Canada and USA but none have focused on qualitative outcomes involving the nurses, doctors and the dentists working in the UK, using formal review criteria for this timeframe. Meta-synthesis of qualitative data using framework synthesis can identify the common recurring themes in a broad context and therefore help policy decisions. This review is limited by the lack of literature on the dental workforce and draws on limited publications in the medical and nursing literature in the UK. It highlights lack of publications, which may be due to publication bias towards workforce research and qualitative research or a lack of research in this area.

Systematic reviews on migration of HRH, in the literature, have focused on trends in workforce migration [57], but not compared the motives across health care professionals. Integration studies have focused on qualitative thematic analyses of lived experiences, particularly in relation to nurses [54, 58, 59] and on doctors [60], but none on dentists. This review is a starting point examining the drivers of migration and integration experiences of the three professions of nursing, medicine and dentistry, which are different in relation to the professional’s control of their work environment, their professional and social identities [17, 20, 27, 28, 36, 38, 59]. Understanding the similarities or variations of migration motives amongst these groups can help in developing bespoke policies for retention, improving job satisfaction and performance, all of which are important to any health system that wants to maximise its HRH potential.

## Conclusion

This review is considered timely as the UK prepares to leave the EU, with implications on recruitment of EU health professionals, whilst the NHS is still reliant on international professionals to meet the workforce deficit. There is a lack of qualitative literature on international doctors’ and dentists’ migration and integration compared with nurses, despite their contributions to the UK workforce. Active recruitment, post graduate training and financial gain act as strong common macro, meso and micro drivers that perpetuates migration into the UK, but the extent to which each of these drivers influence nurses and doctors migration is different.

Integration experiences for international nurses and doctors differed as nurses reported a wider knowledge and skills gap, more multi-level discrimination and less career progression compared with doctors. A better understanding of the migration motives and integration experiences of different health care professions will help form policies that are bespoke and therefore more effective in recruitment and retention, which in turn will help reduce UK’s reliance on international workforce. Understanding the barriers to and facilitators of integration for each of these professions is also important for migrants, employers and policy makers to develop a personalised health care system that can meet the sustainable development goals of the WHO Global workforce strategy. Further research into the dental workforce is clearly required.

## Additional files

Additional file 1: Electronic search sample using PubMed. (DOCX 14 kb)

Additional file 2: Eligibility screening checklist. (PDF 408 kb)

Additional file 3: Quality scoring summary. (XLSX 34 kb)

## Abbreviations

GHWA: Global Health Workforce Alliance
HRH: Human resources for health
HWMPI: Health Worker Migration Policy Initiative
OECD: Organisation for Economic Co-operation and Development
PRISMA: Preferred Reporting Items for Systematic Reviews and Meta-Analyses
WHO: World Health Organization
