# Supplementary material for: Migration motives and integration of international human resources of health in the United Kingdom: systematic review and meta-synthesis of qualitative studies using framework analysis
Source: Hum Resour Health. 2018 Jun 27;16:27. doi: 10.1186/s12960-018-0293-9 (PMC6020357; doi:10.1186/s12960-018-0293-9)
Supplement: Supplementary file 1 — Electronic search sample using PubMed. (DOCX 14 kb) [file 12960_2018_293_MOESM1_ESM.docx]

**Additional file 1: Electronic search sample using PubMed**

| Search on PubMed | Total hits | Selected based on titles |
| --- | --- | --- |
| ***(((((((migration) OR integration) OR adaptation) OR career aspirations) OR job satisfaction) OR discrimination) AND international dental graduates) AND overseas dentists***. Your search for *(((((((migration) OR integration) OR adaptation) OR career aspirations) OR job satisfaction) OR discrimination) AND international dental graduates) AND overseas dentists* retrieved no results.  (((("migration"[All Fields] OR "migration"[All Fields]) OR ("integration"[All Fields] OR "Integration (Amst)"[Journal] OR "integration"[All Fields])) AND (internationally[All Fields] AND trained[All Fields] AND ("nurses"[MeSH Terms] OR "nurses"[All Fields]))) AND (overseas[All Fields] AND ("nurses"[MeSH Terms] OR "nurses"[All Fields]))) AND ("nurses, international"[MeSH Terms] OR ("nurses"[All Fields] AND "international"[All Fields]) OR "international nurses"[All Fields] OR ("foreign"[All Fields] AND "nurses"[All Fields]) OR "foreign nurses"[All Fields]) AND ("2000/01/01"[PubDate] : "2017/01/31"[PubDate])  ((((((("migration"[All Fields] OR "migration"[All Fields]) OR ("integration"[All Fields] OR "Integration (Amst)"[Journal] OR "integration"[All Fields])) OR ("acclimatization"[MeSH Terms] OR "acclimatization"[All Fields] OR "adaptation"[All Fields])) OR ("discrimination (psychology)"[MeSH Terms] OR ("discrimination"[All Fields] AND "(psychology)"[All Fields]) OR "discrimination (psychology)"[All Fields] OR "discrimination"[All Fields])) OR ("job satisfaction"[MeSH Terms] OR ("job"[All Fields] AND "satisfaction"[All Fields]) OR "job satisfaction"[All Fields])) AND (international[All Fields] AND medical[All Fields] AND graduates[All Fields])) AND (overseas[All Fields] AND ("physicians"[MeSH Terms] OR "physicians"[All Fields] OR "doctors"[All Fields]))) AND (("internationality"[MeSH Terms] OR "internationality"[All Fields] OR "foreign"[All Fields]) AND ("physicians"[MeSH Terms] OR "physicians"[All Fields] OR "doctors"[All Fields])) AND ("2000/01/01"[PubDate] : "2017/01/31"[PubDate]) | 17  97  149 | 5  25  18 |
| ***Total*** | 263 | 48 |
