# Supplementary material for: Migration motives and integration of international human resources of health in the United Kingdom: systematic review and meta-synthesis of qualitative studies using framework analysis
Source: Hum Resour Health. 2018 Jun 27;16:27. doi: 10.1186/s12960-018-0293-9 (PMC6020357; doi:10.1186/s12960-018-0293-9)
Supplement: Supplementary file 2 — Eligibility screening checklist. (PDF 408 kb) [file 12960_2018_293_MOESM2_ESM.pdf]

REVIEWER INITIAL: _____________

Eligibility screening checklist

Migration motives and integration of international nursing, medical and dental graduates: a synthesis of qualitative studies

Inclusion criteria	Yes	Group	No	Unclear
Do the participants belong to one or all of these groups? ITNs, IMGs, IDGs?				
Is the study conducted in the UK or reporting about UK				
Study design<br>A. Does it have primary qualitative data or separately reported data for each outcome<br>B. Is the study in English<br>C. Was it published between Jan 2000- Jan 2017				
Do the study outcomes refer to all or one of the following issues:<br>A. Migration motives of the ITNs, IMGs and IDGs<br>B. Integration ITNs, IMGs and IDGs in the destination countries				
Final decision:	Include		Exclude	Discuss
